# Determination of Corneal Biomechanical Behavior *in-vivo* for Healthy Eyes Using CorVis ST Tonometry: Stress-Strain Index

**DOI:** 10.3389/fbioe.2019.00105

**Published:** 2019-05-16

**Authors:** Ashkan Eliasy, Kai-Jung Chen, Riccardo Vinciguerra, Bernardo T. Lopes, Ahmed Abass, Paolo Vinciguerra, Renato Ambrósio Jr., Cynthia J. Roberts, Ahmed Elsheikh

**Affiliations:** ^1^School of Engineering, University of Liverpool, Liverpool, United Kingdom; ^2^St Paul's Eye Unit, Royal Liverpool and Broadgreen University Hospital, Liverpool, United Kingdom; ^3^Department of Biomedical Science, Humanitas University, Rozzano, Italy; ^4^Eye Center, Humanitas Clinical and Research Center, Rozzano, Italy; ^5^Rio de Janeiro Corneal Tomography and Biomechanics Study Group, Rio de Janeiro, Brazil; ^6^Department of Ophthalmology, Federal University of the State of Rio de Janeiro, Rio de Janeiro, Brazil; ^7^Department of Ophthalmology and Visual Science, Department of Biomedical Engineering, The Ohio State University, Columbus, OH, United States; ^8^NIHR Biomedical Research Centre for Ophthalmology, Moorfields Eye Hospital NHS Foundation Trust and UCL Institute of Ophthalmology, London, United Kingdom; ^9^School of Biological Science and Biomedical Engineering, Beihang University, Beijing, China

**Keywords:** cornea, biomechanics, material properties, numerical modeling, finite element modeling

## Abstract

**Purpose:** This study aims to introduce and clinically validate a new algorithm that can determine the biomechanical properties of the human cornea *in vivo*.

**Methods:** A parametric study was conducted involving representative finite element models of human ocular globes with wide ranges of geometries and material biomechanical behavior. The models were subjected to different levels of intraocular pressure (IOP) and the action of external air puff produced by a non-contact tonometer. Predictions of dynamic corneal response under air pressure were analyzed to develop an algorithm that can predict the cornea's material behavior. The algorithm was assessed using clinical data obtained from 480 healthy participants where its predictions of material behavior were tested against variations in central corneal thickness (CCT), IOP and age, and compared against those obtained in earlier studies on *ex-vivo* human ocular tissue.

**Results:** The algorithm produced a material stiffness parameter (Stress-Strain Index or SSI) that showed no significant correlation with both CCT (*p* > 0.05) and IOP (*p* > 0.05), but was significantly correlated with age (*p* < 0.01). The stiffness estimates and their variation with age were also significantly correlated (*p* < 0.01) with stiffness estimates obtained earlier in studies on *ex-vivo* human tissue.

**Conclusions:** The study introduced and validated a new method for estimating the *in vivo* biomechanical behavior of healthy corneal tissue. The method can aid optimization of procedures that interfere mechanically with the cornea such as refractive surgeries and introduction of corneal implants.

## Introduction

The ability to determine corneal biomechanical properties *in-vivo* is of great clinical importance as it can help optimize several treatments and management procedures that interact or interfere mechanically with the eye. Examples include measurement of intraocular pressure (IOP) for effective glaucoma management (Kaushik et al., 2012; Elsheikh et al., 2015), refractive surgery planning (Roberts, 2002; Pepose et al., 2007), keratoconus risk profiling (Ortiz et al., 2007; Ambrósio et al., 2017a), optimization or judging different protocols of collagen cross-linking treatments (Goldich et al., 2012), pre-op evaluation of refractive surgery re-treatment, selection of intracorneal ring implants and even design of soft contact lenses where the mechanical interaction between the lens and the anterior eye is currently not considered.

A main challenge in estimating the corneal biomechanical behavior *in vivo* stems from the difficulty in separating the effects of this behavior from those of the IOP on ocular response to mechanical stimuli. This challenge has made it difficult to produce accurate IOP estimates, that are free of the effects of corneal biomechanics (Liu and Roberts, 2005), and the same challenge exists in determining the tissue's biomechanics that are free of the effects of IOP. Nevertheless, the compound nature of this challenge has meant that finding a solution for either IOP or corneal biomechanics would lead to a solution for the other problem.

What complicates matters further is that the stress-strain behavior of biological tissue, including cornea and sclera, is non-linear (Ethier et al., 2004; Elsheikh et al., 2007), and therefore the tangent modulus (E_t_)—a measure of material stiffness—does not have a constant value, but increases with stress and strain. This effectively means that as the IOP in the eye increases, the stress and strain to which the eye is subjected increases, causing a rise in the tangent modulus. Therefore, the problem is not only that the effects of IOP and corneal biomechanics on eye behavior are difficult to separate; IOP also effects the immediate corneal stiffness.

A positive development toward achieving a solution to this problem was the introduction of the biomechanically-corrected IOP (bIOP) estimates based on the CorVis ST (OCULUS Optikgeräte GmbH; Wetzlar, Germany) output (Elsheikh et al., 2015). The bIOP algorithm was developed using a combination of numerical modeling, experimental and clinical validation (Elsheikh et al., 2010a; Joda et al., 2016), as well as corneal deformation parameters (measured by the CorVis ST) to reduce the effect of stiffness on IOP estimates (Eliasy et al., 2018). With the bIOP shown in earlier studies to be less correlated with the cornea's stiffness parameters than both GAT and the uncorrected CorVis ST IOP (CVS-IOP) measurements (Chen et al., 2018), this study takes the next logic step in providing estimates of the material mechanical behavior.

This step is taken in this study where the emphasis is on an algorithm that can provide an estimate of the whole stress-strain behavior that would, in turn, enable determination of E_t_ under any IOP, and would ultimately be suitable for use in numerical simulation exercises to exploit the benefits of material characterization in clinical applications such as refractive surgery planning or cross-linking therapy optimization.

*Hypothesis: The study was based on the hypothesis that a biomechanical CorVis index can be numerically developed and shown to be almost independent of CCT and IOP but maintained positive correlation with age in healthy patients*.

## Methods

The study relied on numerical models of the full eye globe subjected to both IOP and the air pressure of the CorVis ST. The models enabled simulation of wide ranges of ocular topography, thickness profiles, IOP values and material behavior trends that extend beyond those seen in ophthalmic practice or reported in the literature. The analysis resulted in predictions of corneal deformation and CorVis output parameters for each combination of the input parameters, and these predictions were used to develop an algorithm providing estimates of the tissue's material behavior as a function of the cornea's geometric parameters, the IOP measurement and the CorVis output parameters. The algorithm was then validated by assessing the correlation between its material stiffness predictions and patient age in two clinical datasets, and against earlier results of inflation experiments on *ex-vivo* human eyes (Eliasy et al., 2018).

### Numerical Modeling

**Figure 1:**
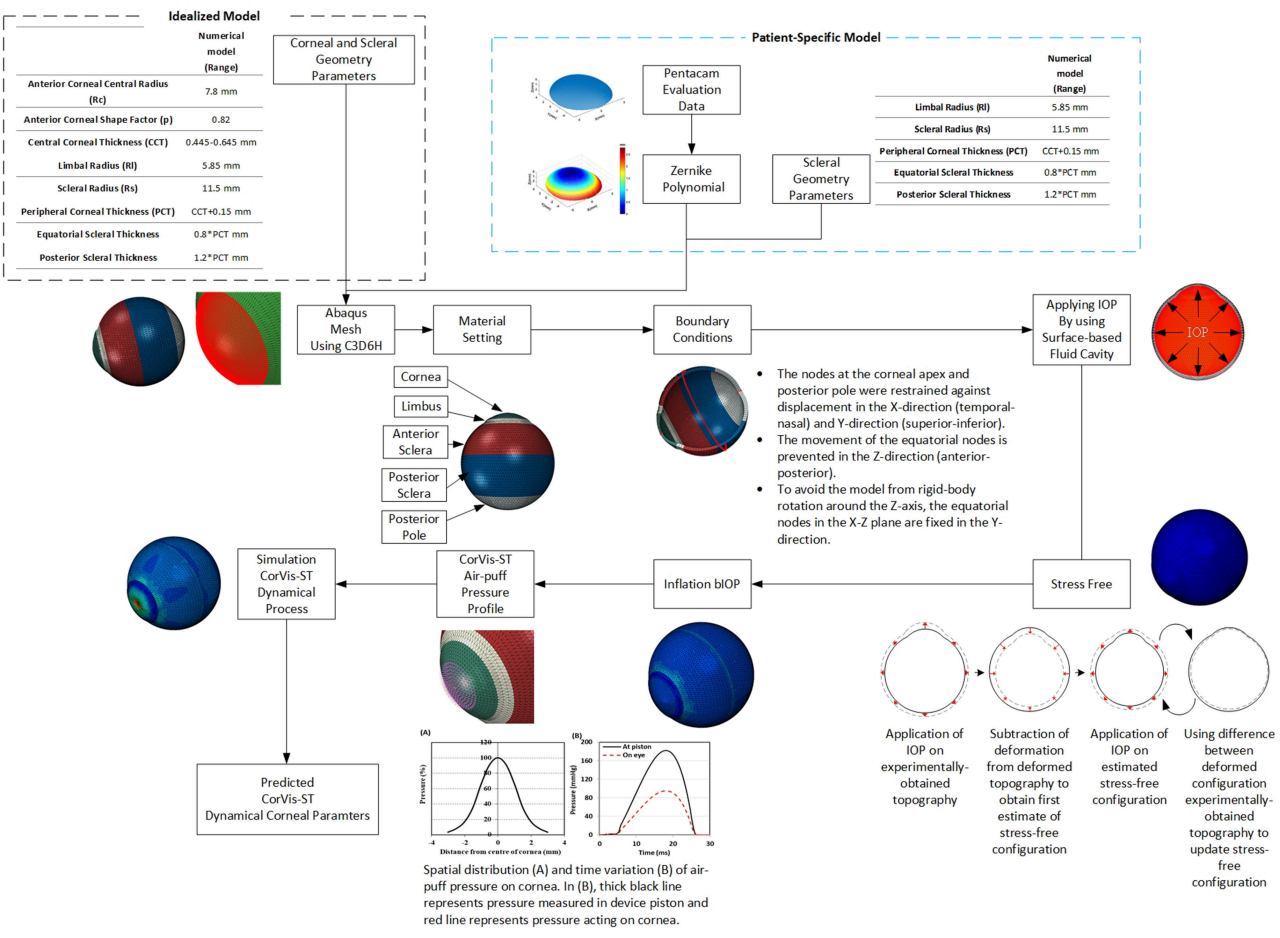
Flowchart is demonstrating the process behind the analysis of built-in-house mesh generator software.

**Figure 2:**
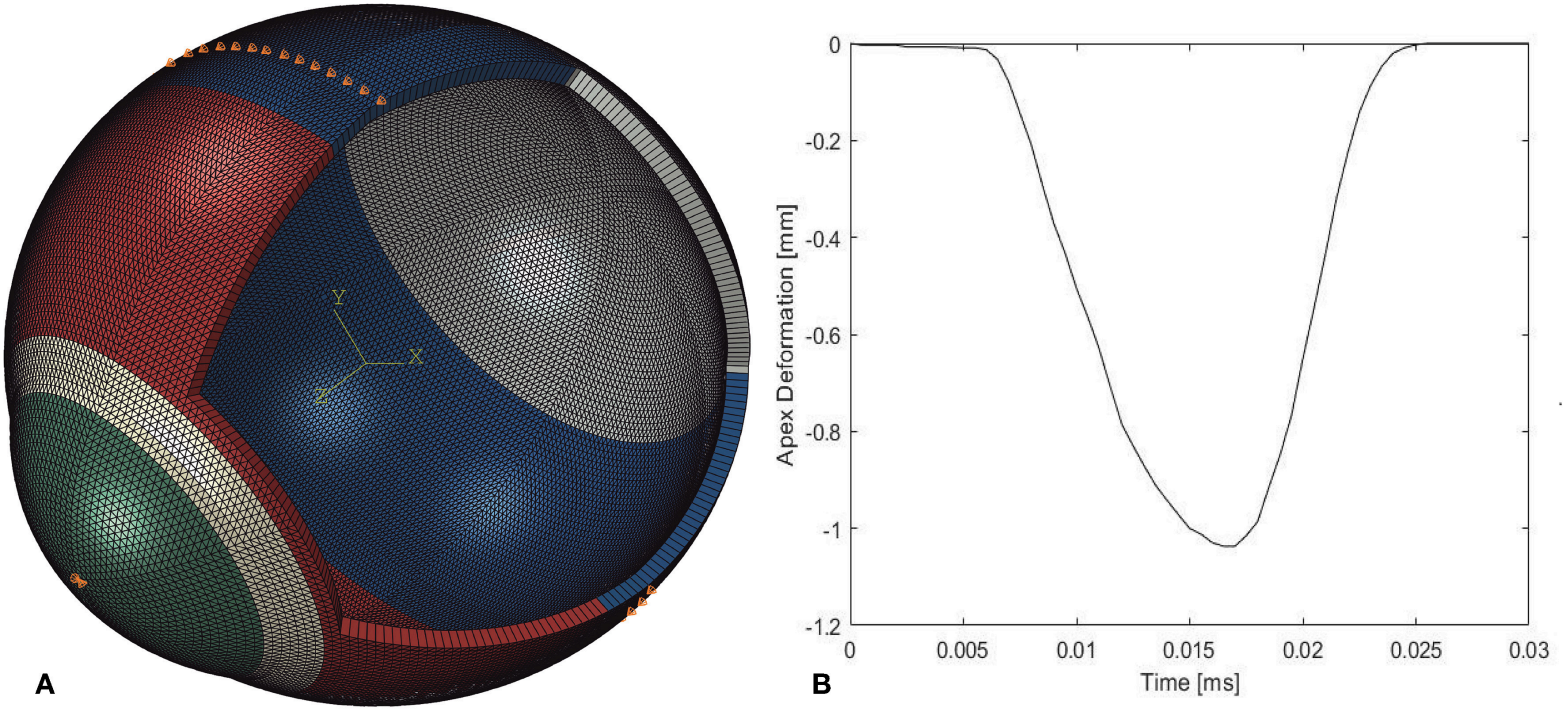
**(A)** A typical finite element model showing the boundary conditions applied at the equator and corneal apex and the four model regions, each with its own material behavior. **(B)** Apex deformation of the numerical model during application of air-puff.

Finite element models of full eye globes were developed by a bespoke ocular mesh-generator software tool [developed in house (Whitford et al., 2015)] and analyzed using Abaqus 6.14 FE solver (Dassault Systèmes Simulia Corp., Providence, RI, USA), Figure 1. The models included 65,712 six-noded, continuum C3D6H elements, connected by 65,716 nodes, and organized in 25 cornea element rings and 124 sclera element rings, Figure 2.

Rigid-body motion of the models was prevented by restricting the equator nodes in the anterior-posterior direction, and the corneal apex in both the superior-inferior and temporal-nasal directions, Figure 2. The models had a fluid cavity filled with an incompressible fluid with a density of 1,000 kg/m^3^ to simulate the aqueous and vitreous and their incompressible behavior (Villamarin et al., 2012). IOP was applied and varied in the model through controlling the pressure in this internal fluid. This technique enabled the internal eye pressure to vary from the initial IOP according to the deformation experienced under the CorVis air pressure. At the start of the analysis, the stress-free form of each model, which corresponded with a state under IOP = 0 mmHg, was reached using an iterative process (Elsheikh et al., 2013) before applying IOP followed by the CorVis air pressure.

The eye model was divided into four regions incorporating the cornea, limbus, anterior sclera, and posterior sclera, with different stress-strain behavior patterns. Third-order, hyper-elastic Ogden models were used to represent the ocular tissue's mechanical behavior as obtained in previous experimental studies where correlation between stress-strain behavior and age was reported (Elsheikh et al., 2010b; Geraghty et al., 2012). Moreover, scleral regional variation in stiffness, with a gradual reduction in stiffness from the limbus toward the posterior pole, was incorporated in the numerical models (Elsheikh et al., 2010a).

### CorVis Simulation

**Figure 3:**
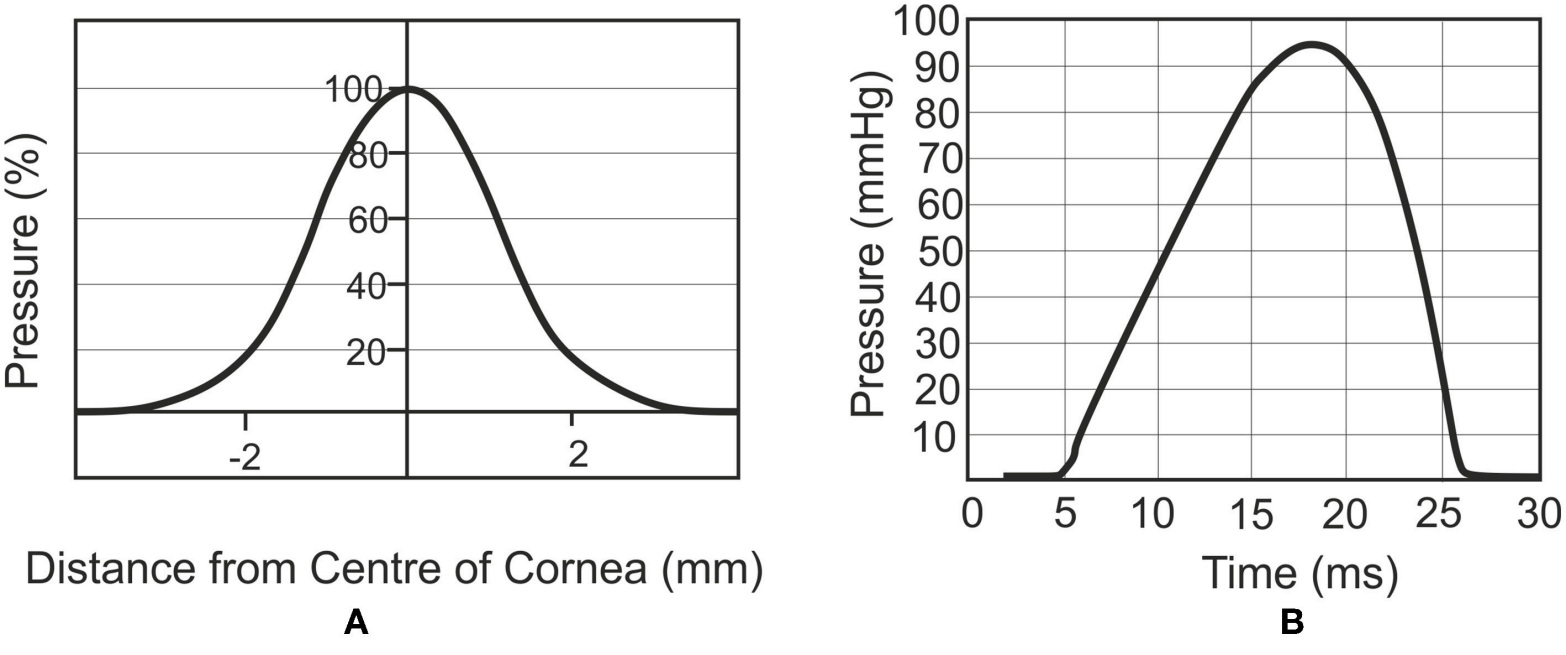
Spatial distribution **(A)** and temporal variation **(B)** of air pressure applied by the CorVis ST on the cornea (Joda et al., 2016).

The air puff of the CorVis was applied on ocular numerical models as per the results of experiments provided by the manufacturer and depicted in Figure 3 (Elsheikh et al., 2009). The results indicated a maximum air pressure of 95 mmHg at corneal apex, reducing away from the apex to a zero value at 4 mm radius. Figure 3B shows the profile of pressure applied by the CorVis on the cornea, which starts with a 5 ms stage with relatively low pressure followed by a fast rise then fall of pressure within approximately a 22 ms period.

### Parametric Study

The numerical models were used in a parametric study that covered wide variations in IOP, geometry and material parameters. IOP varied between 10 and 30 mmHg (in steps of 5 mmHg), covering the values commonly seen in ophthalmic practice, while central corneal thickness (CCT) varied between 445 and 645 microns (in steps of 50 microns). These values of CCT covered and slightly extended beyond the ranges reported in clinical studies, while corneal curvature was fixed at 7.8 mm (Dubbelman et al., 2002; Belin and Khachikian, 2006; Gilani et al., 2013). The peripheral corneal thickness (PCT) was assumed larger than CCT by 150 microns (Avitabile et al., 1997; Ambrósio et al., 2006) with a linear growth in thickness between the two, and in the sclera, the thickness varied linearly from PCT at the limbus, to 80% of PCT at the equator and 120% of PCT at the posterior pole, based on findings of an earlier experimental study (Elsheikh et al., 2010a). The optic nerve head was not simulated in the models as its effect on corneal behavior was expected to be insignificant.

**Figure 4:**
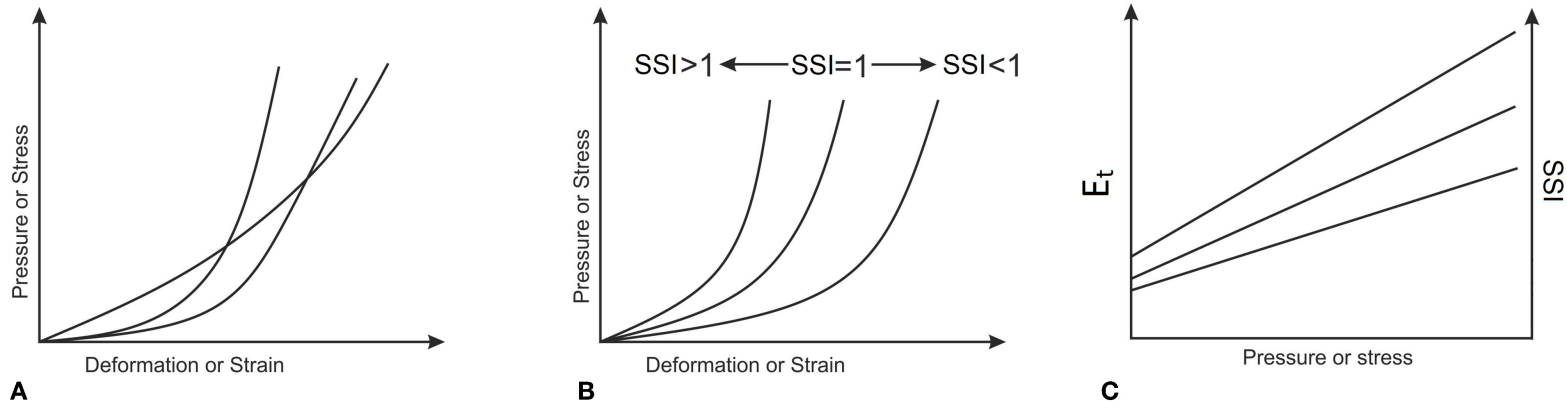
Material biomechanical behavior where **(A)** stress-strain curves intersect, or **(B)** stress-strain curves follow similar patterns. The almost linear variation of E_t_ and SSI with applied pressure or stress, which corresponds to the behavior patterns in **(B)** is depicted in **(C)**.

In order to consider variations in the tissue's material properties, experimental stress-strain behavior obtained in earlier studies (Elsheikh et al., 2010b; Geraghty et al., 2015) by the Biomechanical Engineering Group was assessed and found to follow the similar trends depicted in Figure 4B, rather than the intersecting trends shown in Figure 4A. This feature meant that different stress-strain curves could be obtained from each other while applying a simple stretching factor as a multiplier to all strain values. This factor, called in this study the Stress Strain Index, or SSI, was taken as 1.0 for the average experimental behavior obtained for corneal tissue with age = 50 years (Elsheikh et al., 2010b). Higher values of SSI would then be indicative of higher tissue stiffness, and vice versa.

The average behavior of 50 year old tissue was determined experimentally as (Elsheikh et al., 2010b):

(1) $$\begin{array}{l} \sigma =1.26\times 10^{-3}\times \left({\text{e}}^{102.9\times \varepsilon }-1\right) \end{array}$$

As this σ – ε behavior is approximately exponential, the resulting E_t_ – σ behavior would be almost linear. This feature enabled making the changes in Stress-Strain Index (SSI) proportional to the changes in E_t_ at any stress level as depicted in Figure 4C. The parametric study considered variations in SSI from 0.30 to 3.00, representing a range of stiffness from very soft to very stiff, respectively.

At the end of each simulation, the eye model's deformation under IOP and CorVis ST pressure was recorded and used to predict values of the main CorVis corneal deformation parameters, including the highest concavity radius, maximum deflection, first applanation pressure and first applanation deflection (Nemeth et al., 2013; Roberts et al., 2017).

### Algorithm to Estimate SSI Parameter

The input parameters of the numerical models (CCT, true IOP, SSI) and the output parameters (bIOP and CorVis deformation parameters) were used to derive an algorithm that provides estimates of SSI based on values of CCT, CorVis parameters and bIOP (the biomechanically-corrected IOP taken as a close representation of true IOP). The CorVis parameters were used first to provide values of the stiffness parameter at highest concavity (SP-HC). SP-HC was developed in an earlier study, is currently provided as a CorVis output, and has been shown to be strongly correlated to the cornea's overall stiffness:

(2) $$\begin{array}{r} \text{SP}-\text{HC}=\frac{Adj\text{AP}1-\text{bIOP}}{{\text{Deflection}}_{\max}-{\text{Deflection}}_{A1}} \end{array}$$

where *Adj*AP1 is the pressure measured at first applanation previously quantified using hot wire anemometry (Roberts et al., 2017); Deflection_max_ is the amplitude of corneal apex deflection at the highest concavity; and Deflection_A1_ is the deflection amplitude of corneal apex at first applanation. The least squares method was then used to develop an algorithm to determine SSI as a function of the numerical modeling input and output parameters; CCT, Biop, and SP. The method adopted the objective function:

(3) $$\begin{array}{r} RMS=\min\sqrt{\frac{1}{N}\overset{N}{\underset{i=1}{\sum }}{\left(SSI_{i}^{Algorithm}-SSI_{i}^{Numerical}\right)}^{2}} \end{array}$$

Where *RMS* is the root mean square of the error, *N* is number of data points, *i* is the counter, *SSI* ^*Algorithm*^ is the value obtained from the algorithm, *SSI* ^*Numerical*^ is the value set in the numerical models.

### Clinical Data and Validation

The SSI algorithm was assessed against clinical data obtained from 480 healthy participants enrolled at the Vincieye Clinic in Milan, Italy (Dataset 1, 253 patients) and Corneal Tomography and Biomechanics Study Group—Rio de Janeiro, Brazil (Dataset 2, 227 patients). Institutional review boards at the two institutions ruled that approval was not needed for this record review study. However, ethical approval for using the data in research had been secured at both institutions when the data was collected, anonymized, and used in earlier studies (Vinciguerra et al., 2016; Ambrósio et al., 2017b), before which participants' informed and written consent was secured before collecting the data. Nevertheless, the ethical standards set out in the 1964 Declaration of Helsinki, and revised in 2000, were observed. All patients were evaluated with a complete ophthalmic examination, including the Corvis ST and Pentacam (OCULUS Optikgeräte GmbH; Wetzlar, Germany). All patients were free of any ophthalmic disease, with a Belin/Ambrósio Enhanced Ectasia total deviation index (BAD-D) derived from the Pentacam of < 1.6 standard deviations (SD) from normative values in both eyes. Patients with previous ocular surgery or disease, myopia <-10D, concurrent, or previous glaucoma or hypotonic therapies were excluded.

All Corvis ST exams were acquired by the same experienced technicians with good quality (QS) scores that enabled calculation of all CorVis dynamic corneal response parameters (DCRs). Moreover, a frame-by-frame analysis of the exams, was performed by an independent masked examiner, to ensure quality of each acquisition. Only one eye per patient was randomly included in the analysis to avoid the bias of the relationship between bilateral eyes that could influence the analysis result. Any CorVis readings with visible rotational misalignment in the corneal profile were excluded from the analysis.

The clinical data were used to validate the SSI algorithm via testing the hypothesis that SSI would not be correlated with corneal thickness or IOP but be dependent on age [because of age's correlation with material stiffness (Elsheikh et al., 2010b)].

### *Ex-vivo* Data and Validation

As another form of validation, the correlation between SSI and age that has been established in the two clinical datasets was compared to what had been found in an earlier study involving inflation tests on *ex-vivo* human corneas (Girard et al., 2009; Elsheikh et al., 2010b). The study, which involved 57 corneas tested under inflation conditions with a posterior pressure simulating IOP, resulted in a stress-strain relationship of the form:

(4) $$\begin{array}{r} \sigma =A\left[e^{B\varepsilon }-1\right] \end{array}$$

Where σ = stress, ε = strain, A = 1.26 × 10^−3^, and B = 0.0013 age^2^ + 0.013 age + 99. Both parameters of A and B are dimensionless. Differentiating Equation 1 with respect to the strain leads to:

(5) $$\begin{array}{r} E_{t}=\frac{d\sigma }{d\varepsilon }=AB\;e^{B\varepsilon }=B\;\left(\sigma +A\right) \end{array}$$

where E_t_ = tangent modulus. At the specific case with age = 50 years (at which SSI = 1.0), B = 102.9. Since the ratio between E_t_ at any age and E_t_ at age = 50 equals the ratio between SSI at this age and SSI at age 50 years, which is 1.0, therefore SSI at any age x can be determined from:

(6) $$\begin{array}{r} \frac{{\text{SSI}}_{\text{age x}}}{{\text{SSI}}_{50}=1.0}=\frac{{\text{E}}_{\text{t}}\left(\text{age x}\right)}{{\text{E}}_{\text{t}}\left(\text{age }50\right)} \end{array}$$

This value of SSI, based on *ex-vivo* results and given in terms of age, has been compared to the values of SSI obtained from analysis of the *in vivo* results, obtained from the two clinical datasets.

## Statistical Analysis

Statistical analyses were carried out using IBM SPSS Statistics 24. Data were expressed as mean, standard deviation and range. Pearson correlation analysis was performed to study the relationships of corneal thickness (CCT), age and IOP with the SSI parameter. In this analysis, *p* values smaller than 0.05 were considered to be indicative of statistical significance.

## Results

### Stress-Strain Index (SSI) Algorithm

The least squares method was used to develop an algorithm that can estimate the value of the SSI parameter based on the numerical modeling input and output parameters CCT, bIOP, and SP-HC. The method resulted in a minimum RMS error of ±3% when the algorithm took the form:

(7) $$\begin{array}{l} \text{SSI}=f({\text{a}}_{1}+{\text{a}}_{2}{\text{C}}_{1}+{\text{a}}_{3}{\text{C}}_{2}+{\text{a}}_{4}{\text{C}}_{1}^{2}+{\text{a}}_{5}{\text{C}}_{1}{\text{C}}_{2}+{\text{a}}_{6}{\text{C}}_{2}^{2}+{\text{a}}_{7}{\text{C}}_{1}^{3}\\ \;+{\text{a}}_{8}{\text{C}}_{1}^{2}{\text{C}}_{2}+{\text{a}}_{9}{\text{C}}_{1}{\text{C}}_{2}^{2}+{\text{C}}_{2}^{3}+\text{ln}(\text{SP}-\text{HC})) \end{array}$$

where C_1_ = CCT/545, C_2_ = bIOP/20, ln(SP-HC) the natural logarithm of the stiffness parameter at highest concavity, and a_1_-a_9_ constants determined by fitting Equation 7 to the numerical input and output values, Table 1.

**Table 1:** Values of constants a_1_–a_9_ used in Equation (7).

**SSI**	**a_1_**	**a_2_**	**a_3_**	**a_4_**	**a_5_**	**a_6_**	**a_7_**	**a_8_**	**a_9_**
0.3	−3.094	5.249	8.982	0.248	−8.423	−2.416	−0.443	1.704	2.198
0.5	−7.731	22.224	7.699	−17.455	−8.806	−1.515	5.361	2.852	1.471
0.7	0.440	0.387	4.723	2.974	−5.498	−0.403	−1.200	2.386	0.404
0.8	4.509	−10.507	3.013	12.998	−3.028	0.017	−4.315	1.583	0.002
0.9	7.603	−17.995	0.764	18.971	0.888	0.297	−5.826	−0.114	−0.259
1.0	8.047	−18.217	−0.500	18.236	3.236	0.395	−5.235	−1.242	−0.336
1.5	−8.355	30.668	1.754	−30.649	0.651	−0.519	11.572	−1.163	0.653
2.0	−3.101	16.284	−0.219	−18.494	4.480	−0.208	9.073	−3.482	0.508
2.5	4.677	−9.969	3.607	10.742	−1.410	−1.504	−1.413	−1.463	1.804
3.0	6.842	−16.245	3.244	17.519	−4.064	0.222	−3.391	1.251	0.092

*CCT, central corneal thickness; SSI, stress-strain index*.

### Clinical Validation

#### Dataset 1 (Milan)

**Figure 5:**
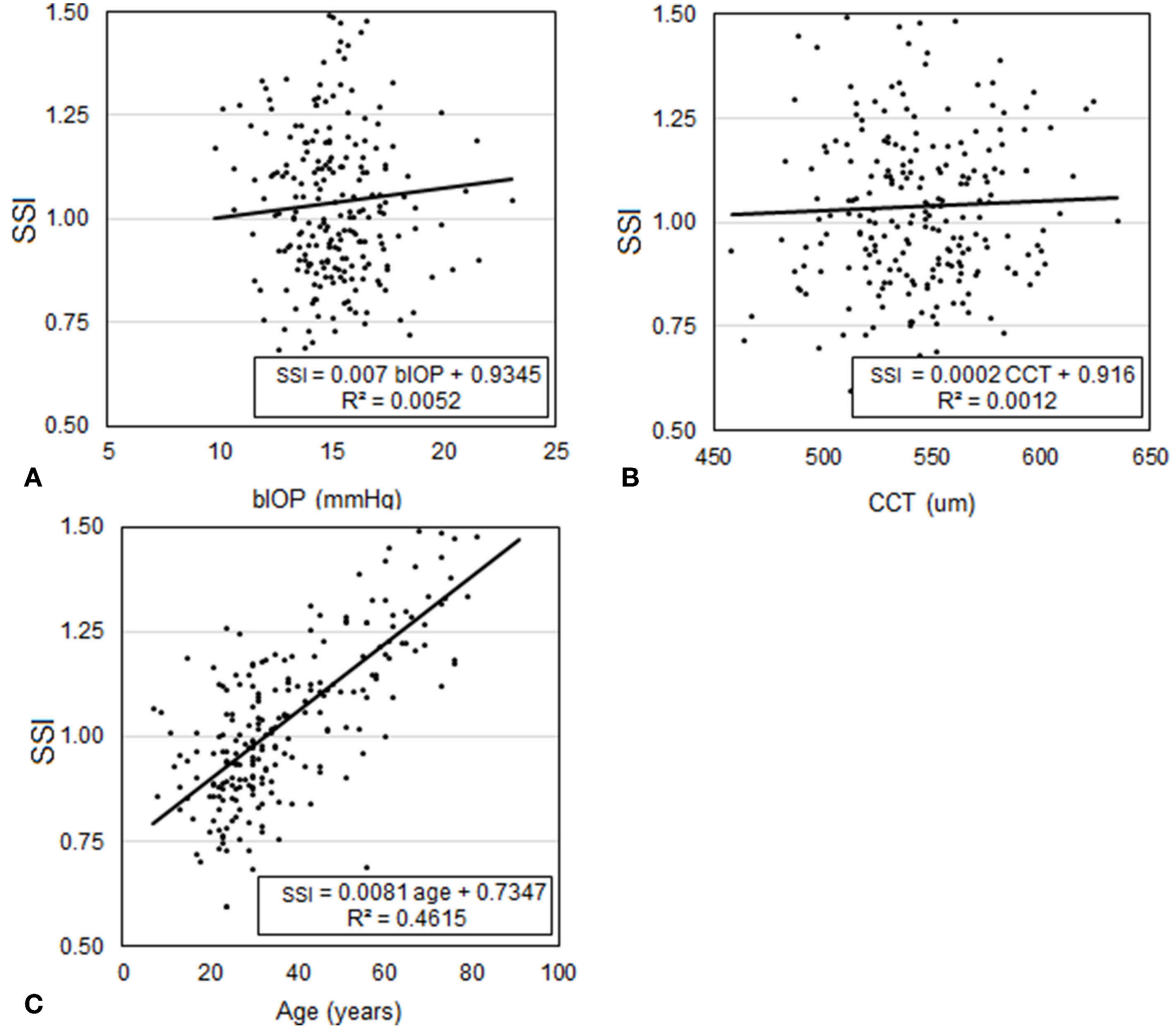
Assessment of the correlation in Dataset 1 between SSI and each of **(A)** bIOP, **(B)** CCT, and **(C)** age.

Participants included in Dataset 1 had a mean age of 43.3 ± 16.6 (8–87) years, CCT of 539.3 ± 33.2 (454–629) microns, and bIOP of 14.3 ± 2.6 (7.7–29.3) mmHg. Analysis of CCT, bIOP, age and SSI values confirmed the hypothesis that SSI was not dependant on CCT (*p* = 0.792) or IOP (*p* = 0.745) but significantly correlated with age (*P* < 0.01), Figure 5. Statistical analysis was performed using Pearson correlation for bIOP and CCT as the data were normally distributed and with Spearman's rho correlation for age where the data were not normally distributed.

#### Dataset 2 (Rio)

**Figure 6:**
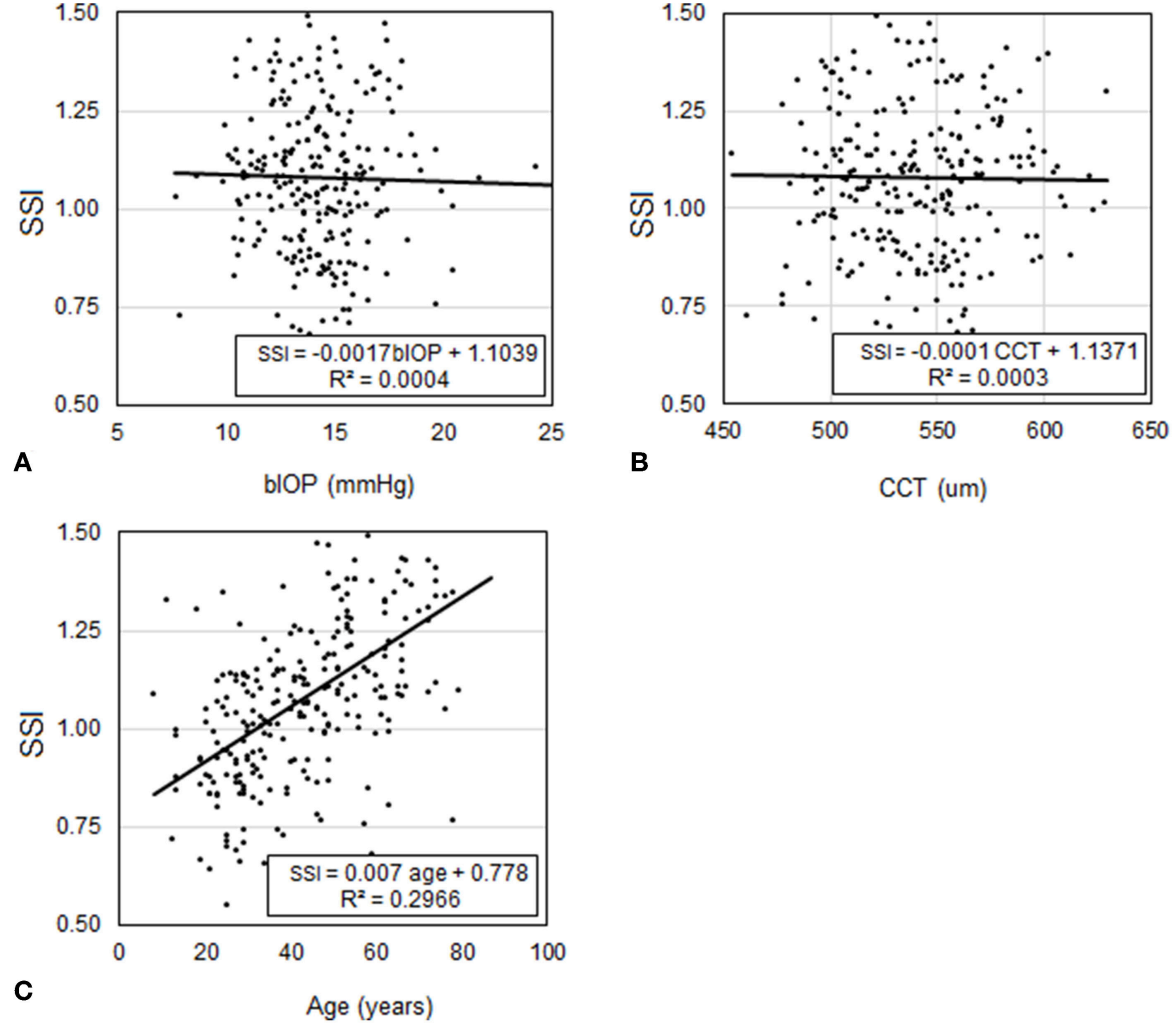
Assessment of the correlation in Dataset 2 between SSI and each of **(A)** bIOP, **(B)** CCT, and **(C)** age.

In Dataset 2, participants had a mean age of 39.9 ± 16.7 (7–81) years, CCT of 543.8 ± 29.4 (454–621) microns, and bIOP of 14.5 ± 2.3 (9.8–24.3) mmHg. Similar to Dataset 1, the analysis showed that SSI was not dependant on CCT (*p* = 0.599) or bIOP (*p* = 0.281), but was significantly correlated with age (*P* < 0.01), Figure 6. Statistical analysis was performed using Pearson correlation with bIOP and CCT and Spearman's rho correlation with age for the reasons described above.

#### Combined Datasets

In order to increase the statistical power of results, the analysis was repeated while combining the two datasets. In this analysis, participants had a mean age of 40.6 ± 17.1 (7–87) years, CCT of 541.5 ± 32.43 (454–629) microns, and bIOP of 14.7 ± 2.4 (7.7–29.3) mmHg. Similar to the analysis conducted above, statistical comparisons showed that SSI was not dependant on CCT (*p* = 0.999) or bIOP (*p* = 0.480), but was significantly correlated with age (*p* < 0.01). The analysis was performed using Pearson correlation with bIOP and CCT and Spearman's rho correlation.

### Validation Against *ex-vivo* Inflation Test Results

**Figure 7:**
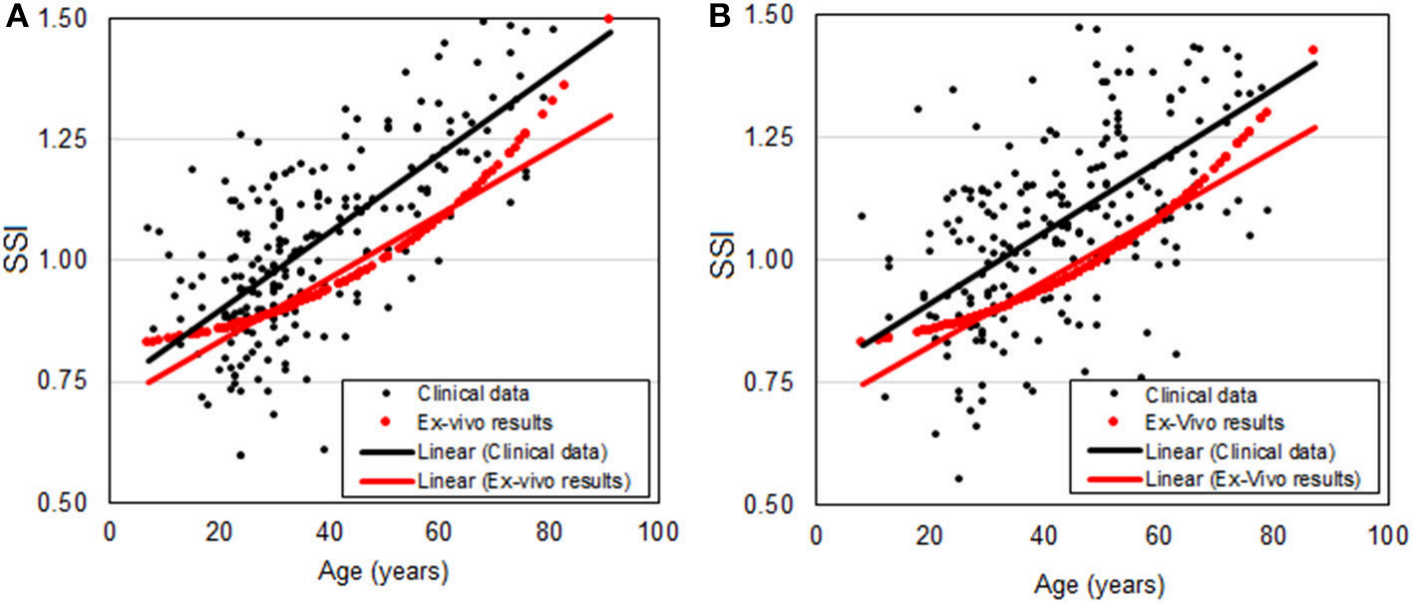
Relationship between SSI and age based on *in-vivo* clinical data (black dots and a trend black line) and *ex-vivo* inflation test results (red dots) for **(A)** Milan dataset and **(B)** Rio dataset.

The relationship between SSI and age plotted in Figures 5C, 6C for Datasets 1 and 2, respectively, is re-plotted in Figure 7 and compared with the relationship based on *ex-vivo* inflation test results (Elsheikh et al., 2007). The comparison shows close correlation between the two relationships with the differences being 0.09 ± 0.20 (*p* < 0.01) and 0.10 ± 0.21 (*p* < 0.01) for Datasets 1 and 2, respectively. Statistical analysis was performed using Spearman's rho correlation as the data were not normally distributed.

## Discussion

This paper attempts to address a long-standing challenge related to the *in-vivo* measurement of corneal biomechanics, and in doing so it attempts to overcome two major obstacles. First, the nonlinear nature of the tissue behavior makes it necessary to determine the whole stress-strain behavior, rather than a tangent modulus value which would be valid only at a particular level of stress or strain. This obstacle was overcome through an observation that stress-strain relationships obtained earlier for *ex-vivo* ocular tissue had similar trends that saw almost proportional decreases in strain with increases in tissue age. By taking the behavior of corneal tissue at age 50 years as the benchmark, at which the new SSI parameter was assumed equal to 1.0, other stress-strain relationships for stiffer or softer material could be derived by multiplying the strain values by the relevant value of the SSI parameter.

The second challenge stems from the effect of IOP and corneal thickness on corneal deformation under the action of internal or external mechanical actions. However, while the effect of corneal thickness on overall behavior is large, it can be estimated and removed as the thickness and its effect can be measured and excluded accurately. On the other hand, IOP presents a more difficult challenge since IOP measurement methods—through tonometry—are affected by corneal stiffness, creating a challenging dilemma with the stiffness affecting IOP measurement and IOP affecting corneal mechanical behavior, which is used to estimate the stiffness. In this study, this challenge was addressed through consideration of a Corvis parameter—the stiffness parameter, SP-HC—which is more strongly correlated with corneal stiffness than IOP. For brevity, SSI is intended to be independent of IOP and corneal geometry and is needed to estimate the material stiffness, hence it is not the same as Stiffness Parameter (SP).

The new SSI algorithm was generated based on predictions of corneal behavior using finite element (FE) numerical modeling simulating the effects of IOP and Corvis ST air puff. The algorithm was then validated through assessment of its correlation with IOP, CCT and age in two large clinical datasets. As expected, SSI was found to be independent of both IOP (*p* = 0.745 in Dataset 1, *p* = 0.281 in Dataset 2) and CCT (*p* = 0.792 in Dataset 1, *p* = 0.599 in Dataset 2), while being correlated with age (*p* < 0.01 in Dataset 1, *p* < 0.01 in Dataset 2), which, in turn, was found earlier (in an experimental study on *ex-vivo* human eyes) to be strongly associated with material stiffness (Elsheikh et al., 2007).

Another validation exercise was conducted by comparing the relationship between SSI and age established in the two datasets against the results of the earlier *ex-vivo* study (Elsheikh et al., 2010b). The comparisons showed there were no significant differences between the relationships (*p* < 0.01 in both Datasets 1 and 2).

The introduction of the SSI algorithms in clinical practice could enable customization of the diagnosis and management of ocular diseases and allow optimization of clinical procedures that either interact or interfere mechanically with the eye. With successful validation, SSI could help in identifying eyes with keratoconus, possibly increasing the sensitivity and specificity of indexes such as the Corvis Combined Biomechanical Index (CBI) (Vinciguerra et al., 2016) or the Tomography and Biomechanical Index (TBI) (Ambrósio et al., 2017b). Moreover, it could help in the detection of patients with higher risk or susceptibility for ectasia development or progression after refractive surgery and could aid in surgery planning (Ambrósio et al., 2017a).

Glaucoma management could also benefit from the accurate measurement of corneal biomechanics (Kaushik et al., 2012). Among the factors that influence the accuracy of IOP measurement is the corneal tissue's mechanical stiffness, and therefore quantifying the stiffness using the SSI algorithm could lead to improvements in IOP measurement and possibly better glaucoma management outcomes (Liu and Roberts, 2005).

There have been previous attempts to measure corneal mechanical properties *in vivo*. These included the Corneal Hysteresis (CH) and Corneal Resistance Factor (CRF) produced by the Ocular Response Analyzer (ORA) (Luce, 2005), and the Stiffness Parameter (SP) (Roberts et al., 2017) by the CorVis. These parameters were correlated with the diagnosis of keratoconus and showed significant increases after collagen cross-linking (CXL) (Bak-Nielsen et al., 2014) but could not provide measures of material behavior that were separate from the effects of geometry and IOP. Another attempt is the elastic modulus provided by Brillouin microscopy (Scarcelli et al., 2013), which, while related to the cornea's material stiffness, is not compatible with the nonlinear stress-strain behavior that means the tissue does not have a unique modulus, but has a tangent modulus, which increases gradually with stress or applied pressure.

The SSI algorithm developed in this study is only suitable for corneas with normal topography. Corneas with keratoconus or ectasia, in which the geometry does not match the numerical models used in this work, will be treated separately in a future publication. Earlier work demonstrated the importance of including the ciliary muscles in simulations of corneal mechanical response to both IOP and external air pressure, but not the iris or the lens (Whitford, 2016). Earlier studies also confirmed the much lower stiffness of the retina relative to the ocular outer tunic (cornea and sclera) (Chen et al., 2010) and for this reason, it was not included in the numerical models.

In conclusion, we introduced in this study a new method for estimating the material behavior of healthy corneal tissue that can aid in optimization of procedures that interact or interfere mechanically with the cornea.

## Ethics Statement

The SSI algorithm was assessed against clinical data obtained from 480 healthy participants enrolled at the Vincieye Clinic in Milan, Italy (Dataset 1, 253 patients) and Corneal Tomography and Biomechanics Study Group—Rio de Janeiro, Brazil (Dataset 2, 227 patients). Institutional review boards at the two institutions ruled that approval was not needed for this record review study. However, ethical approval for using the data in research had been secured at both institutions when the data was collected, anonymized, and used in earlier studies (Vinciguerra et al., 2016; Ambrósio et al., 2017b), before which participants' informed and written consent was secured before collecting the data. Nevertheless, the ethical standards set out in the 1964 Declaration of Helsinki, and revised in 2000, were observed. All patients were evaluated with a complete ophthalmic examination, including the Corvis ST and Pentacam (OCULUS Optikgeräte GmbH; Wetzlar, Germany). All patients were free of any ophthalmic disease, with a Belin/Ambrósio Enhanced Ectasia total deviation index (BAD-D) derived from the Pentacam of < 1.6 standard deviations (SD) from normative values in both eyes. Patients with previous ocular surgery or disease, myopia < -10D, concurrent, or previous glaucoma or hypotonic therapies were excluded.

## Author Contributions

K-JC conducted the research. AsE and RV drafted the manuscript, interpreted data, performed statistical analysis. AsE validated the findings and assisted in supervision. RV provided clinical data. AA, PV, RA, CR, and BL performed interpretation of data and provided clinical data. AhE developed the concept, design the project and supervised the entire research and secured funding. All authors reviewed the manuscript and provided final approval.

### Conflict of Interest Statement

RA, PV, RV, CR, and AhE are consultants for OCULUS Optikgeräte GmbH. AhE has received research funding from OCULUS Optikgeräte GmbH. The remaining authors declare that the research was conducted in the absence of any commercial or financial relationships that could be construed as a potential conflict of interest.
